# The association between fetal-stage exposure to the China famine and risk of diabetes mellitus in adulthood: results from the China health and retirement longitudinal study

**DOI:** 10.1186/s12889-018-6134-x

**Published:** 2018-10-26

**Authors:** Zhenghe Wang, Zhiyong Zou, Zhongping Yang, Yanhui Dong, Jieyun Song, Bin Dong, Jun Ma, Luke Arnold

**Affiliations:** 1 School of Public Health & Institute of Child and Adolescent Health, Peking University, No 38 Xue Yuan Road, Haidian District, Beijing, 100191 China; 2 Population Health & South Western Sydney Primary Health Network, Sydney, NSW Australia

**Keywords:** Starvation, Diabetes mellitus, Fetal nutrition disorders, Sex characteristics

## Abstract

**Background:**

The associations of famine exposure with diabetes risk in adulthood are still unclear. This study aimed to explore the association between famine exposure in early life and risk of diabetes in adulthood.

**Methods:**

A total of 4138 subjects were selected from the data of the China Health and Retirement Longitudinal Study (CHARLS) 2011–2012. Diabetes was diagnosed as fasting plasma glucose (FPG) ≥7.0 mmol/L, glycated haemoglobin (HbA_1C_) > 6.5%, or self-reported diabetes. Birthdates of subjects were used to categorize famine exposure groups. The association of fetal-stage famine exposure with diabetes risk in adults was assessed using logistics regression model.

**Results:**

The prevalence of diabetes in the non-exposed, fetal-stage exposed, infant-stage exposed, and preschool-stage exposed groups were 9.0, 13.6, 12.7 and 10.8%, respectively. Compared with the age-balanced control group, the fetal-stage exposed group was associated with the elevated risk of diabetes in later life after adjusting for covariates (OR = 1.37; 95%CI: 1.09–1.72; *P* = 0.008). Stratified analysis showed that the association between prenatal famine exposure and diabetes risk in adulthood was comparable between severely affected areas and less severely affected areas (*P* for interaction =0.153).

**Conclusions:**

Famine exposure in fetal stages was associated with the elevated diabetes risk in adults, which could be the critical periods for relative intervention.

**Electronic supplementary material:**

The online version of this article (10.1186/s12889-018-6134-x) contains supplementary material, which is available to authorized users.

## Background

Cardiovascular disease has become an important public health challenge across the world, including China [1, 2]. As a major contributor to the burden of disease, diabetes mellitus increased sharply with the prevalence rise from less than 1% in 1980 to 11.6% in 2010 in Chinese adults [3]. Apart from China’s emerging economy, shifting lifestyle, and ageing, several studies have reported that severe malnutrition exposure in early life is linked with adverse health outcomes in later life [4, 5]. Exploring this association in humans is inappropriate as it violates the medical ethics in conducting research with human beings, however, natural historical famine provides us a unique opportunity to investigate the associations between severe food shortage in early life and adverse health consequences in later life.

The Great Chinese Famine happened between 1959 and 1961. Not only did it result in 30 million premature deaths, but also left millions of famine victims [6–8]. In the last decades, several studies have found that famine exposure in early-life significantly increased the risk of non-communicable diseases in adulthood, including hypertension [9–11], dyslipidemia [12], metabolic syndrome [13, 14], and fatty liver [15]. Several studies also assessed the relation between exposure to famine in early life and the risk of developing hyperglycemia or diabetes in the Chinese population [16–19]. However, only one study reported that fetal-stage exposure to the Chinese Famine increased the risk of adulthood hyperglycemia, however did not observe a significant association of famine exposure and diabetes risk [19]. Using occupational or regional data, others researchers also explored this association in the Chinese population, but the effect of age difference between exposed groups and non-exposed group was not controlled in those studies [16–18]. A recent meta-analysis revealed that the association between famine exposure and the risk of adult diabetes was not consistent with the use of different controls, suggesting that the age difference between famine exposure and non-exposed groups could explain the main effect of famine [20]. Further research is needed to clarify that association with a comparable age between exposed and non-exposed groups [20, 21].

The current study used the national survey data from the China Health and Retirement Longitudinal Survey (CHARLS) to explore the association between fetal-stage famine exposure and adulthood diabetes risk compared with an age-balanced controls.

## Methods

### Participants

Participants in the current study were selected from the baseline survey of the CHARLS in 2011–2012. The CHARLS survey was a national large epidemiological survey with a focus on health and retirement of the Chinese elder population. In order to broadly representing the entire elder population of mainland China, the survey selected 17,708 participants from 10,257 households with at least one family member aged ≥45 years old from 450 villages or communities in 28 provinces [22]. In the current study, 4317 participants born between from 1956 to 1964 were selected to participate in the study. After excluding 189 participants whose birthplaces were not within the current provinces and 44 participants who were missing information on diabetes, 4138 participants were involved in the final analysis. This study was a secondary analysis of the identified CHARLS public data. The Medical Ethics Committee of Peking University granted the current study exemption from review. ZZ and ZW received permission from the CHARLS team to use this.

### Classification

The Great Chinese Famine lasted for approximately 3 years between 1959 and 1961. The participants were categorized into four groups according to their year of birth. We defined participants born between October 1st 1962 and September 30th 1964 as the non-exposed group; participants born between October 1st 1959 and September 30th 1961 as the fetal-stage exposed group; participants born between January 1st 1958 and December 31st 1958 as the infant-stage exposed group; and participants born between January 1st 1956 and December 31st 1957 as the preschool-stage exposed group. Consistent with the previous study, the excess mortality in famine periods (1959–1961) compared with mortality in pre-famine periods (1956–1958) was used to reflect the severity of famine exposure [12]. Excess mortality of 50.0% was used as a threshold to categorize all provinces into severely affected areas (Excess mortality ≥50.0%) and less severely affected areas (Excess mortality < 50.0%) [12].

### Diagnosis of diabetes mellitus

The fasting plasma was collected to measure the concentration of the fasting plasma glucose (FPG) using enzymatic colorimetric analysis. HbA1c was also tested using the boronate affinity High Performance Liquid Chromatography (HPLC). Participants were diagnosed as having diabetes mellitus if they had either a fasting plasma glucose reading of ≥7.0 mmol/L, Hb1Ac > 6.5%, or self-reported diabetes [23].

### Covariates

The CHARLS baseline questionnaire was used to collect the information on social demographic characteristics, lifestyle, and health behaviors. The short form International Physical Activity Questionnaire (IPAQ-SF) was used to assess physical activity, which was also used to categorize the physical activity into 3 groups (light, moderate, and vigorous physical activity) [24]. Smoking status was categorized into never smoking, former smoking (who smoked more than 100 cigarettes in life and had quit smoking more than a years ago) and current smoking groups (who smoked at least one cigarette per day in the last year). Alcohol consumption was categorized into never, former (consumed alcohol more than once per week more than a year ago, but may not drink currently) and current (consumed alcohol at least once per week in the last year) groups. The highest educational attainment of participants and their parents was categorized into four groups, including primary school and below, junior school, high school, and college or above. Overweight and obesity were defined using the body mass index (BMI) ≥24.0 kg/m^2^ in line with the recommendation for Chinese adults [25]. A participant’s age was calculated as the difference between detection date and birthdate.

### Statistical analysis

The computer-aided personal survey (CAPI) system was used to collect the data. Statistical Package for Social Science (SPSS) version 20.0 (SPSS Inc. Chicago, IL) was used to perform the statistical analysis.

Analysis of Variance (ANOVA) was used to test variance of continuous variables (including age, BMI, FPG, and Hb1Ac) between four groups, and the Dunnett-*t* test was used to conduct the multiple comparisons between three exposed groups (fetal-stage, infant-stage, and preschool-stage exposed groups) with non-exposed group. The differences of categorical variables, including sex, diabetes prevalence, physical activity level, smoking, drinking, education attainments, and overweight/obesity prevalence, were detected by *Chi*-square test.

Because there are no overlaps in the birth years among the 4 famine exposed groups, the adjustment for age may not improve the estimation [21]. To control the effect of age difference on the association, we generated an age-balanced control group of fetal-exposed group (mean age: 52.2 vs 51.8 years) by combining non-exposed group (pre-famine) and infant- and preschool-stage exposed group (post-famine), The diabetes risk between fetal-exposed group and age-balanced control group was assessed with logistic model. While Model 1 did not adjust for any covariate, Model 2 adjusted for sex and BMI, and Model 3 further adjusted for smoking status, drinking status, physical activity level, the highest educational attainments of participants and their parents. To explore the effect of severity, sex and BMI on the association, we conducted a stratified analysis. The interaction terms were also tested. A *P* < 0.05 with two sides was considered as statistically significant.

## Results

The basic characteristics of participants are shown in Table 1. A total of 4138 participants were enrolled into the present study. The sample sizes of the non-exposed, fetal-stage, infant-stage, and preschool-stage exposed groups were 1536, 832, 519, and 1251, respectively. Diabetes prevalence of the non-exposed, fetal-stage, infant-stage, and preschool-stage exposed groups were 9.0, 13.6, 12.7 and 10.8%, respectively. Though the mean levels of FPG and Hb1Ac% were not significantly different (*P* > 0.05), diabetes prevalence in fetal-stage and infant-stage exposed groups were significantly higher than that in the non-exposed group (13.6 and 12.7% vs. 9.0%, *P* < 0.05). Additionally, the fetal-stage exposed group had higher educational attainment and overweight/obesity prevalence than the non-exposed group and participants in infant-stage exposed group had a higher BMI level than preschool-stage exposed group. Preschool–stage exposed group had higher current smoking rates and mean ages than the non-exposed group.

**Table 1:** Basic characteristics of study population according to the Chinese famine exposure

	Non-exposed group	Fetal exposed group	Infant exposed group	Preschool exposed group	*P* value
Birth date	10/1/1962–9/30/1964	10/1/1959–9/30/1961	1/1/1958–12/31/1958	1/1/1956–12/31/1957	
N	1536	832	519	1251	
Women, n (%)	803(52.3)	439 (52.8)	241 (46.4)	615 (49.2)	0.035
Diabetes, n (%)	139 (9.0)	113 (13.6)^*^	66 (12.7)^*^	135 (10.8)	0.004
Age (Mean ± SD, years)	48.84 ± 0.66	51.83 ± 0.66	54.00 ± 0.00	55.49 ± 0.50	< 0.001
Physical activity level, n (%)					0.215
Light	1007 (65.6)	540 (64.9)	371 (71.5)	821 (65.6)	
Moderate	306 (19.9)	166 (20.0)	88 (17.0)	257 (20.5)	
Vigorous	223 (14.5)	126 (15.1)	60 (11.6)	173 (13.8)	
Smoking, n (%)					0.018
Never	1048 (68.2)	554 (66.6)	330 (63.3)	775 (62.0)	
Former	80 (5.2)	36 (4.3)	28 (5.4)	78 (6.2)	
Current	408 (26.6)	242 (29.1)	161 (31.0)	398 (31.8)	
Drinking, n (%)					0.613
Never	956 (62.2)	514 (61.8)	320 (61.7)	791 (63.2)	
Former	154 (10.0)	81 (9.7)	54 (10.4)	100 (8.0)	
Current	426 (27.7)	237 (28.5)	145 (27.9)	360 (28.8)	
Own education, n (%)					< 0.001
Primary school and below	647 (42.1)	356 (42.8)	276 (53.2)	730 (58.4)	
Junior	579 (37.7)	224 (26.9)	145 (27.9)	313 (25.0)	
High school	251 (16.3)	225 (27.0)	88 (17.0)	188 (15.0)	
College and above	59 (3.8)	27 (3.2)	10 (1.9)	20 (1.6)	
Parents’ education, n (%)					0.667
Primary school and below	1367 (89.0)	744 (89.4)	468 (90.2)	1141 (91.2)	
Junior	87 (5.7)	47 (5.6)	24 (4.6)	57 (4.6)	
High school	65 (4.2)	30 (3.6)	20 (3.9)	45 (3.6)	
College and above	17 (1.1)	11 (1.3)	7 (1.3)	8 (0.6)	
Overweight/obesity, n (%)	543 (48.1)	290 (47.0)	170 (44.0)	402 (41.3)	0.012
BMI (Mean ± SD, kg/m^2^)	24.21 ± 3.61	24.29 ± 4.22	23.72 ± 3.85	23.69 ± 3.79	0.002
FPG (Mean ± SD, mg/dl)	107.15 ± 34.11	110.13 ± 39.38	110.81 ± 38.78	108.19 ± 30.70	0.246
Hb1Ac%(Mean ± SD,%)	5.21 ± 0.84	5.26 ± 0.88	5.30 ± 0.88	5.23 ± 0.80	0.272

*Abbreviations*: *SD* standard deviation, *BMI* body mass index, *FPG* fasting plasma glucose

^*^Compared with the non-exposed cohort, *P* < 0.05

Table 2 presents the associations between fetal-stage famine exposure and risk of diabetes in adulthood using different control groups. Compared with the age-balanced control group, the fetal-stage exposed group was associated with increased risk of diabetes (OR = 1.37; 95%CI: 1.09–1.72; *P* = 0.007). Partial adjustments had same results as fully adjusted models (*P* < 0.05). However, famine exposure was negatively related with diabetes risk when controls were limited to infant exposed (OR = 0.92; 95%CI: 0.66–1.27; *P* = 0.427) or pre-school exposed (OR = 0.77; 95%CI: 0.59–1.01; *P* = 0.052) through the relationships were not statistically significant.

**Table 2:** The risk of diabetes in later life following exposure to famine using different control groups

Fetal-exposed group		Control groups			*OR*(95%*CI*)
No. cases/ sample size	Mean age (years)	Control groups	No. cases/ sample size	Mean age (years)	
Total					
113/832	51.8 ± 0.7	Non-exposed	139/1536	48.8 ± 0.6	1.58 (1.21–2.06)^**^
		Infant-exposed	66/519	54.0 ± 0.5	0.92 (0.66–1.27)
		Preschool-exposed	135/1251	55.5 ± 0.4	0.77 (0.59–1.01)
		Age balanced	340/3306	52.2 ± 3.2	1.37 (1.09–1.72)^**^
Severe area					
57/471	51.8 ± 0.7	Non-exposed	75/1010	48.8 ± 0.7	1.70 (1.18–2.45)^**^
		Infant-exposed	44/343	54.0 ± 0.5	1.05 (0.69–1.61)
		Preschool-exposed	86/819	55.5 ± 0.5	0.87 (0.61–1.25)
		Age balanced	205/2172	52.2 ± 3.2	1.33 (0.97–1.82)
Less severe area					
56/361	51.9 ± 0.7	Non-exposed	64/526	48.9 ± 0.7	1.33 (0.90–1.96)
		Infant-exposed	22/176	54.0 ± 0.5	0.77 (0.45–1.31)
		Preschool-exposed	49/432	55.5 ± 0.5	0.66 (0.43–1.01)
		Age balanced	135/1134	52.2 ± 3.2	1.36 (0.97–1.91)
Male					
55/393	51.9 ± 0.7	Non-exposed	72/733	48.9 ± 0.7	1.51 (1.03–2.20)^*^
		Infant-exposed	32/278	54.0 ± 0.5	0.76 (0.47–1.24)
		Preschool-exposed	62/636	55.5 ± 0.5	0.66 (0.44–0.97)^*^
		Age balanced	166/1647	52.3 ± 3.2	1.47 (1.05–2.04)^*^
Female					
58/439	51.8 ± 0.6	Non-exposed	67/803	48.8 ± 0.7	1.72 (1.18–2.50)^**^
		Infant-exposed	34/241	54.0 ± 0.5	1.01 (0.63–1.60)
		Preschool-exposed	73/615	55.5 ± 0.5	0.86 (0.59–1.26)
		Age balanced	174/1659	52.0 ± 3.2	1.35 (0.98–1.86)
BMI < 24 kg/m2					
43/423	51.8 ± 0.7	Non-exposed	56/807	48.8 ± 0.7	1.51 (0.99–2.29)
		Infant-exposed	25/285	54.0 ± 0.5	0.87 (0.52–1.47)
		Preschool-exposed	58/701	55.5 ± 0.5	0.84 (0.55–1.29)
		Age balanced	139/1793	52.3 ± 3.2	1.34 (0.93–1.92)
BMI ≥ 24 kg/m2					
70/409	51.9 ± 0.7	Non-exposed	83/729	48.8 ± 0.7	1.64 (1.16–2.31)^**^
		Infant-exposed	41/234	54.0 ± 0.5	1.00 (0.65–1.53)
		Preschool-exposed	77/550	55.5 ± 0.5	0.75 (0.52–1.07)
		Age balanced	201/1513	52.0 ± 3.2	1.38 (1.02–1.86)^*^

*Abbreviations*: *OR* odds ratio, *CI* confidence interval, *NO*. numbers

All the analysis adjusted for gender, BMI, smoking status, drinking status, physical activity level, the highest education attainments of participants and their parents. ^*^ *P* < 0.05; ^**^ *P* < 0.01

The associations between the fetal-exposed group and adult diabetes risk were further stratified by severity, sex and BMI. The associations were comparable between severely affected areas (OR = 1.33; 95%CI: 0.97–1.82; *P* = 0.054) and less severely affected areas (OR = 1.36; 95%CI: 0.97–1.91; *P* = 0.061). After stratifying by sex, the association was stronger in males (OR = 1.47; 95%CI: 1.05–2.04; *P* = 0.024) than that in females (OR = 1.35; 95%CI: 0.98–1.86; *P* = 0.053). When stratified by BMI, among participants who were categorized as overweight/obese, the adult risk of diabetes in the fetal-exposed group was significantly higher than that in the age-balanced control group (OR = 1.38; 95%CI: 1.02–1.86; *P* = 0.036), but not among participants who were categorized as normal weight (OR = 1.34; 95%CI: 0.93–1.92; *P* = 0.069). In addition, a significant interaction between BMI and fetal-exposed group was observed (*P*_interaction_ = 0.003). However, consistent associations were not found for areas and sex groups (*P* > 0.05).

## Discussion

The current study used the data of a Chinese national representative survey and found that famine exposure in the fetal period was closely associated with risk of diabetes in adulthood. These results suggest that the fetal period could be the crucial period for determining future diabetes risk.

Previous studies conducted in China have reported the association between early life famine exposure and risk of diabetes in adulthood. However, the results are inconsistent. Two studies based on the data from the Chinese National Nutrition and Health Survey [19] and the Dongfeng-Tongji cohort [17] found famine exposure in early life was related to the hyperglycemia in adulthood. However, whether famine exposure is associated with risk of diabetes is unclear. Using the Survey on Prevalence in East China for Metabolic Diseases and Risk Factors, Wang [18] reported that participants who suffered severe famine exposure in fetal-stage had a significantly higher risk of diabetes than those who suffered less severe famine exposure. In addition, a recent meta-analysis suggested that age variation between famine exposed and unexposed groups could explain most effects of famine. For a more reliable estimate, this study used an age appropriate control group to examine the long-term effect of the Chinese Famine on survivors’ health. Adding to previous studies, we observed that fetal-stage exposure to the Great Chinese Famine was associated with an obviously enhanced diabetes risk in adulthood, which increased by 37% compared with the age-balanced control group. Additionally, the famine exposure seems to has a ‘protective’ effect on diabetes when the controls are limited to infant-exposed or pre-school exposed because of age differences. These associations support that fetal-stage exposed to famine has an adverse influence on the burden of diabetes in adulthood [26, 27].

Several mechanisms may explain the association between famine exposure in the fetal period and later risk of diabetes. Firstly, epigenetic changes might play an essential role. Several Dutch famine studies have observed that famine exposure during the fetal period could change methylation levels of genes in biological pathways involved in growth and metabolic function, including pancreatic beta cell functioning (*SMAD7*) and insulin signaling (*INSR*) [28, 29]. Secondly, severe malnutrition during early life could alter the expression level of certain genes related to growth and metabolic. A study conducted in rodents has found that perinatal severe protein restriction permanently changes the expression of gene clusters involved in regulating insulin signaling and nutrient sensing [30]. In addition, another study found that severe starvation exposure lead to poor development of pancreatic beta cell mass and function in rats, which might persist into adulthood [31]. Moreover, poor intrauterine nutritional condition may influence the development of skeletal mass and increase the risk of insulin resistance in later life [32].

The association between the famine exposure in fetal period and diabetes risk seems to be stronger among males than females in our study. Similar sex disparity was also observed in our previous study, which found that males were associated with the higher risks of early life famine exposure related chronic lung diseases [33]. We speculate that the sex difference might be linked with the sensitivity response to famine exposure between sexes. Males are more susceptible than females to adverse effects of famine exposure in the perinatal periods [34, 35]. More study is needed to clarify the sex gap in this association.

In the current study, we also found that overweight and obesity in later life were associated with higher diabetes risk in adulthood. Similar association was also described in previous studies conducted in China and Netherlands [19, 27]. The mismatch between perinatal poor nutritional environment and postnatal rich environment could partly explain the famine related risk of diabetes [32]. Our results suggest that both improving intrauterine nutrition environment and controlling weight in the postnatal period are important for ameliorating glycemic profile.

The unexpected finding was the associations between famine exposure and diabetes risk was not different between severely affected areas and less severely affected areas. These results indicated that within area variations in famine severity may exceed between area differences in famine severity. This means that the areas employed may be too large to adequately define famine severity at the local level.

Additionally, we did not observe significantly difference for mean level of FPG between famine exposure and non-exposed groups, whereas diabetes did. It was inconsistent with previous study of Li, which reported that famine exposure group had a higher mean level of FPG, whereas diabetes did not [19]. We speculated that age of participants and hypoglycemic therapy may contribute to the difference. The mean age of participants in Li’s study is 10 years younger than that in the current study. Participants with hypoglycemic therapy in the current study might be more than that in the Li’s study due to higher diabetes prevalence (13.6% vs. 1.70%). Thus, the mean FPG level of exposure group in the current study could be decreased.

Several limitations of the current study should be noted. Firstly, the survivor bias could be a main limitation of our study. The China famine caused approximately 30 million deaths and many subjects with severe metabolic and structural disorders in the perinatal period may have died in the famine. Hence, the survivors could be relatively healthier subjects. The bias is acceptable in the present study because it attenuates the effect of famine exposure, and underestimates magnitude of the association we presented. Secondly, we cannot accurately divide fetal and infant exposures because the famine did not start exactly from 1959. Thus, there may be participants who were exposed to famine in infant stage may be included in the fetal-stage exposure group. This misclassification could decrease the real association between fetal-stage famine exposure and risk of diabetes in adults, which have been validated by performing similar regression analysis within population without excluding these participants (Additional file 1: Table S1 and Table S2 in supplemental materials). To reduce this misclassification, the current study excluded the participants whose birthdates from January 1st 1959 to September 30th 1959. Third, we did not collect objective indicators, including personal famine exposure, birth weight, and birth length, in this study, which could reflect the effect of famine on a personal level. A study with more individual information collected is warranted. In addition, though the age-balanced control group was used, the difference of age between age-balanced control group and fetal-infant-famine exposed group remains. However, that age difference is small (less than 1 year) and unlikely to change our results significantly.

## Conclusions

Exposure to the Great Chinese Famine exposure in early life is associated with elevated diabetes risk in adults. Future studies conducted in other population are required to confirm our findings.

## Additional file

Additional file 1: **Table S1.** Basic characteristic of study population without excluding subjects who born during 1/1/1959–9/30/1959 and 10/1/1961–9/30/1962 according to Chinese famine exposure. **Table S2.** Associations between famine exposure and diabetes prevalence risk in population without excluding subjects who born during 1/1/1959–9/30/1959 and 10/1/1961–9/30/1962, odds ratio (95% confidence interval). Abbreviations: *OR*, odds ratio; *CI*, confidence interval. Age-balanced control group as the reference group. Model 1 did not adjust for any covariate. Model 2 adjusted for gender and BMI, Model 3 further adjusted for smoking status, drinking status, physical activity level, parents’ and their own the highest education attainments. (DOCX 17 kb)

## Abbreviations

ANOVA: Analysis of variance
BMI: Body mass index
CAPI: Computer-aided personal survey
CHARLS: China Health and Retirement Longitudinal Study
CI: Confidence interval
FPG: Fasting plasma glucose
HbA_1C_: Glycated haemoglobin;
HPLC: High performance liquid chromatography
IPAQ-SF: International Physical Activity Questionnaire- short form
OR: Odds ratio
SPSS: Statistical package for social sciences
