# Supplementary material for: The association between fetal-stage exposure to the China famine and risk of diabetes mellitus in adulthood: results from the China health and retirement longitudinal study
Source: BMC Public Health. 2018 Oct 26;18:1205. doi: 10.1186/s12889-018-6134-x (PMC6204016; doi:10.1186/s12889-018-6134-x)
Supplement: Supplementary file 1 — Table S1. Basic characteristic of study population without excluding subjects who born during 1/1/1959–9/30/1959 and 10/1/1961–9/30/1962 according to Chinese famine exposure. Table S2. Associations between famine exposure and diabetes prevalence risk in population without excluding subjects who born during 1/1/1959–9/30/1959 and 10/1/1961–9/30/1962, odds ratio (95% confidence interval). Abbreviations: OR, odds ratio; CI, confidence interval. Age-balanced control group as the reference group. Model 1 did not adjust for any covariate. Model 2 adjusted for gender and BMI, Model 3 further adjusted for smoking status, drinking status, physical activity level, parents’ and their own the highest education attainments. (DOCX 17 kb) [file 12889_2018_6134_MOESM1_ESM.docx]

**Table S1.** Basic characteristic of study population without excluding subjects who born during 1/1/1959-9/30/1959 and 10/1/1961-9/30/1962 according to Chinese famine exposure

| Variables | Non-exposed  group | Fetal-exposed  group | Infant-exposed  group | Preschool-exposed  group |
| --- | --- | --- | --- | --- |
| Birth date | 10/1/1962-9/30/1964 | 10/1/1959-9/30/1962 | 1/1/1958-9/30/1959 | 1/1/1956-12/31/1957 |
| N | 1,536 | 1,431 | 821 | 1,251 |
| Diabetes (%) | 8.8 | 10.9 | 10.8 | 10.9 |

| **Table S2.** Associations between famine exposure and diabetes prevalence risk in population without excluding subjects who born during 1/1/1959-9/30/1959 and 10/1/1961-9/30/1962, odds ratio (95% confidence interval) | | | |
| --- | --- | --- | --- |
|  | *OR* | *95%CI* | *P* |
| Model 1 | 1.10 | 0.90-1.34 | 0.342 |
| Model 2 | 1.09 | 0.89-1.33 | 0.373 |
| Model 3 | 1.07 | 1.84-1.51 | 0.412 |

Abbreviations: *OR*, odds ratio; *CI*, confidence interval.

Age-balanced control group as the reference group.

Model 1 did not adjust for any covariate. Model 2 adjusted for gender and BMI, Model 3 further adjusted for smoking status, drinking status, physical activity level, parents’ and their own the highest education attainments.
